# Sickness absence due to common mental disorders in young employees in Sweden: are there differences in occupational class and employment sector?

**DOI:** 10.1007/s00127-021-02152-3

**Published:** 2021-08-12

**Authors:** Emma Björkenstam, Magnus Helgesson, Klas Gustafsson, Marianna Virtanen, Linda L. Magnusson Hanson, Ellenor Mittendorfer-Rutz

**Affiliations:** 1 Department of Clinical Neuroscience, Division of Insurance Medicine, Karolinska Institutet, 171 77 Stockholm, Sweden; 2 Department of Neuroscience, Uppsala University, Uppsala, Sweden; 3 Department of Community Health Sciences, Fielding School of Public Health and California Center for Population Research, University of California Los Angeles, Los Angeles, CA USA; 4 School of Educational Sciences and Psychology, University of Eastern Finland, Joensuu, Finland; 5 Stress Research Institute, Stockholm University, Stockholm, Sweden

**Keywords:** Occupational class, Common mental disorder, Sickness absence, Cohort, Sweden, Employment, Occupation, Young adults, Epidemiology

## Abstract

**Background:**

A large proportion of sickness absence (SA) in young adults is due to common mental disorders (CMDs). Still studies on CMD-related SA in young workers are lacking, especially studies for those employed in the private sector. The current study investigated the associations between sector of employment, occupational class and SA due to CMDs. In addition, associations between type of employment branch and SA due CMDs within each sector were examined.

**Methods:**

This population-based longitudinal cohort study included 663,583 employees, 19–29 years, residing in Sweden in 2009. Employment sector (i.e., private/public) and occupational class (non-manual/manual workers) were measured in 2009. Risk estimates of SA due to CMDs, between 2010 and 2016, were calculated as Hazard Ratios (HR) with 95% confidence intervals (CI), using Cox regression analysis.

**Results:**

Sector of employment was associated with SA due to CMDs, such that public sector workers had an elevated risk when compared with private sector employees (adjusted HR: 1.31 (95% CI 1.29–1.33). Moreover, manual workers had a slightly elevated risk for SA due to CMDs compared to non-manual workers. Within the private sector, in both manual and non-manual workers, those employed in education and health and social services evidenced the highest rates and risks of SA due to CMDs.

**Conclusion:**

Sector of employment and occupational class play a role in SA due to CMDs in young employees. These findings should be considered when identifying high-risk groups for SA in the young working population.

**Supplementary Information:**

The online version contains supplementary material available at 10.1007/s00127-021-02152-3.

## Introduction

Over the past decades, research has shown that the rates of common mental disorders (CMDs), i.e., depressive, anxiety and stress-related disorders, are high in young adults in many Western countries, including Sweden [1]. According to recent statistics from the World Health Organization (WHO), CMDs account for the largest proportion of mental disorders in children and adolescents [2]. Almost 20% of the population in the WHO European Region aged 10–19 years are estimated to have a mental disorder, of which CMDs accounts for over 40% [2].

CMDs are characterized by having an early age of onset [3], recurrent episodes as well as high levels of comorbid disorders, including substance use disorders and musculoskeletal diseases [3, 4].

Studies have further shown that CMDs in young age may have a potential negative effect on academic attainment, and subsequently on work opportunities [5], as CMDs can significantly reduce the individual’s work ability [6–9]. The loss of productivity associated with reduced work capacity is a very large burden to the individual and the society, if individuals face marginalization at the labor market in early age. In young adults in Sweden, the labor-market situation has become more problematic over the past decade, with youth unemployment rates higher than the OECD average [1]. Recent reports from Norway and Denmark have further shown an alarming increase in sickness absence (SA) in young adults [10], and particularly SA due to CMDs [6]. Mental disorders, and CMDs in particular, are now also the most common reason for SA in Sweden in younger ages [6]. Although several studies have shown that SA due to CMDs is far more common in a group of young adults who are not employed [1], studies on prevalence and incidence rates of SA due to CMDs in young adults who are employed are lacking [11]. The literature is even more limited regarding SA among young workers employed in the private sector. This is somewhat surprising, as up to 80% of young employees in Sweden work in the private sector [12]. Moreover, previous research focusing on differences in SA between labor market sectors, such as private and public sectors is scarce [13–15], especially in young employees.

Two contradictory theoretical viewpoints have been put forward either claiming differences or similarities regarding organizational and management structures as well as psychosocial work stress in public and private sectors [16]. Likewise, studies have found or failed to find differences regarding the psychosocial work environment in these two sectors [17]. Those studies finding differences found that job stress was higher in the public sector. It can, therefore, by hypothesized that SA due to CMDs might be higher in the public compared to the private sector, as an adverse psychosocial work environment has been linked to sickness absence due to these disorders [18]. Studies on these associations are, however, lacking to date.

Within the public sector, those employed in certain occupational groups, such as health care professionals, have been pointed out as having a markedly elevated risk for any SA [10, 19] and SA due to CMDs in particular [20, 21]. Within the private sector on the other hand, a higher risk of SA has been found in employees working in service occupations, whereas lower SA rates have been observed within business and finance professionals [19]. Still studies examining whether similar associations in these sectors also can be found in young workers are lacking to date. With respect to SA, identifying high-risk groups of professionals is of importance when targeting individuals at risk, and to increase our understanding of the determinants of SA due to CMDs.

Occupational class is also associated with physical and psychosocial working conditions, that may affect health and work. Occupational class is often characterized as non-manual and manual, the latter often having higher risk of work disability due to CMDs [21–23]. A recent Finnish study examined the magnitude of occupational class differences in all-cause SA in younger adults in the public sector and found that the SA rates in both women and men were highest in manual workers [24]. Whether similar differences for SA due to CMDs also hold for young employees in the private sector has been less studied to date. Research on the relationship between employment sector and SA due to CMDs is also restricted, especially regarding the younger working population.

The current register-based study used a large cohort of nearly 665,000 employed individuals between the ages of 19 and 29 years in Sweden, to investigate: (i) the associations between sector of employment, occupational class, and SA due to CMDs; and (ii) within each sector, the associations between different types of employment branches and SA due to CMDs.

## Materials and methods

### Study population

The study population was defined from the Longitudinal Integration Database for Health Insurance and Labor Market Studies (LISA) register [25]. This register contains data from the labor market and from the educational and social sectors. We included all individuals, aged 19–29 years residing in Sweden on December 31st, 2009 (*n* = 1,310,264). Only those classified as being in employment in the register in 2009 were included (*n* = 758,846). Those with incomplete or missing information on occupational class (*n* = 93,708, 12.3%) were excluded. Finally, we excluded individuals who were on DP in 2009 (*n* = 1,555, 0.2%). The final study population comprised 663,583 individuals, of whom 79% were working in the private sector.

We used the unique (de-identified) Swedish personal identity number to link information from several population-based registers. The National Patient Register (NPR) includes information on inpatient care since 1987 and for specialized outpatient care since 2001. Diagnoses in NPR are coded according to the International Classification of Diseases version 10 (ICD-10). The Cause of Death Register (CDR) comprises information on all deaths of Swedish residents since 1952. Finally, the Micro-data for analyses of the social insurance (MiDAS) register covers detailed data on SA and DP from 1994 onwards.

### Sector of employment and occupational class

Sector of employment, i.e., private or public sector in which the individuals worked in 2009 was obtained from LISA. We used the Swedish Standard Classification of Occupations, obtained from LISA in 2009 [25], to categorize [26] and study manual and non-manual employees separately. We further categorized individuals according to the branches in which they worked in 2009. Here we used the Swedish Standard Industrial Classification (acronym SNI), which is based on EU’s recommended standard NACE Rev.2, the European Classification of Economic Activities [27]. More specifically, the following seven categories were studied: Industry occupations (including agriculture, forestry and fishing; mining and quarrying; manufacturing; electricity, gas, steam and air conditioning supply; water supply; sewerage etc.); Service occupations (including information and communication; financial and insurance activities; real estate activities etc.); Wholesale and retail trade; Accommodation and food service activities; Transportation (including occupations within transportation and storage); Construction; Education, and Health and social services. In the analyses, those with missing (*n* = 4755) were treated as a separate group.

### Sickness absence (SA)

In Sweden, all residents aged 16–65 years who have income from work, unemployment benefits, parental benefits or student benefits are entitled to sickness benefits from the Social Insurance Agency (SIA), if unable to work due to disease or injury. Sickness benefits amount up to 80% of lost income. For those employed, the employer usually pays for the first 14 days of an SA spell. Thus, data on most of the short SA spells are not available in the MiDAS register, from which we obtained all diagnosis-specific sick-leave spells during the follow-up period 2010–2016. SA due to CMDs were defined as having at least one sick-leave spell with a main diagnosis for major depressive disorders (code according to the International Classification of Diseases version 10, ICD-10: F32–33), phobic anxiety disorders (ICD-10: F40), other anxiety disorders (ICD-10: F41), obsessive–compulsive disorders (ICD-10: F42) and reaction to severe stress and adjustment disorders (ICD-10: F43). In addition, we examined long-term sickness absence (LTSA) due to CMDs, defined as having a number of net SA days > 90 days during follow-up and short-term absence (≤ 90 days).

### Confounders

Several demographic characteristics with known associations to both work factors, SA and CMDs were considered as potential confounders. These included age, sex, highest attained educational level, family situation, type of residential area, country of birth and all-cause LTSA in 2009 (please see Table 1 for information on categorization of the confounders). We also took into consideration psychiatric morbidity at baseline (defined as inpatient or specialized outpatient care with a main diagnosis for mental disorder in 2009 (ICD-10: F00–F99), as well as somatic morbidity in 2009 (defined as inpatient or specialized outpatient care with a main diagnosis for somatic disease) (defined in Table 2). Missing values in any confounder were grouped as separate categories in the multivariate analyses.

**Table 1:** Cohort characteristics stratified by sector of employment and occupational class, in individuals aged 19–29 years residing in Sweden in 2009. Absolute numbers and column percent

Cohort characteristics^a^	Private sector			Public sector		
	All	Manual workers	Non-manual workers	All	Manual workers	Non-manual workers
All	522,137	400,140	121,997	141,446	84,114	57,332
Sociodemographic factors						
Sex						
Women	221,367 (42)	164,564 (41)	56,803 (47)	105,881 (75)	64,503 (77)	41,378 (72)
Men	300,770 (58)	235,576 (59)	65,194 (53)	35,565 (25)	19,611 (23)	15,954 (28)
Mean age (years, SD)	24.7 (3.0)	24.2 (3.0)	26.3 (2.5)	25.2 (3.0)	24.1 (3.1)	26.7 (2.1)
Country of birth						
Sweden	467,437 (90)	357,486 (89)	109,951 (90)	125,109 (88)	74,061 (88)	51,048 (89)
Other Nordic	3255 (1)	1987 (0)	1268 (1)	900 (1)	389 (0)	511 (1)
EU25 (except Denmark, Finland and Sweden)	7422 (1)	5243 (1)	2179 (2)	2150 (2)	687 (1)	1463 (3)
The rest of the world	43,982 (8)	35,389 (9)	8593 (7)	13,267 (9)	8966 (11)	4301 (8)
Missing	41 (0)	35 (0)	6 (0)	20 (0)	11 (0)	9 (0)
Education (years)						
Compulsory school (≤ 9)	44,686 (9)	40,882 (10)	3804 (3)	7035 (5)	6695 (8)	340 (1)
High school (10–12)	332,753 (64)	291,895 (73)	40,858 (33)	66,069 (47)	60,785 (72)	5284 (9)
College or university (> 12)	141,002 (27)	64,579 (16)	76,423 (63)	67,763 (48)	16,388 (19)	51,375 (90)
Missing	3696 (1)	2784 (1)	912 (1)	579 (0)	246 (0)	333 (1)
Family situation						
Married/living with partner without children^b^	16,622 (3)	10,068 (3)	6554 (5)	6268 (4)	2350 (3)	3918 (7)
Married/living with partner with children^b^	72,936 (14)	55,755 (14)	17,181 (14)	29,928 (21)	17,816 (21)	12,112 (21)
Single/divorced/separated/widowed without children^b^	382,798 (73)	288,613 (72)	94,185 (77)	91,115 (64)	51,325 (61)	39,790 (69)
Single/divorced/separated/widowed with children^b^	7499 (1)	6292 (2)	1207 (1)	4331 (3)	3566 (4)	765 (1)
Children (≤ 20 years)^b^	42,281 (8)	39,411 (10)	2870 (2)	9804 (7)	9057 (11)	747 (1)
Missing	1 (0)	1 (0)	0 (0)	0 (0)	0 (0)	0 (0)
Type of residential area						
Big city area	221,528 (42)	151,602 (38)	69,926 (57)	48,556 (34)	26,037 (31)	22,519 (39)
Intermediate (> 90,000 inhabitants)	179,864 (34)	144,477 (36)	35,387 (29)	55,630 (39)	32,575 (39)	23,055 (40)
Small (rural municipalities)	120,745 (23)	104,061 (26)	16,684 (14)	37,260 (26)	25,502 (30)	11,758 (21)

^a^In 2009

^b^Living at home

**Table 2:** Work- and health-related characteristics stratified by sector of employment and occupational class, in individuals aged 19–29 years residing in Sweden in 2009. Absolute numbers and column percent

Work and health-related characteristics	Private sector			Public sector		
	All	Manual workers	Non-manual workers	All	Manual workers	Non-manual workers
All	522,137	400,140	121,997	141,446	84,114	57,332
Work-related characteristics^a^						
Employment branch						
Industry occupations	85,698 (16)	67,504 (17)	18,194 (15)	290 (0)	210 (0)	80 (0)
Service occupations	158,012 (30)	89,631 (22)	68,381 (56)	24,970 (18)	8373 (10)	16,597 (29)
Wholesale and retail trade; Accommodation and food service activities	148,857 (29)	132,254 (33)	16,603 (14)	243 (0)	227 (0)	16 (0)
Transportation	38,517 (7)	33,997 (8)	4520 (4)	241 (0)	104 (0)	137 (0)
Construction	51,548 (10)	47,565 (12)	3983 (3)	1,017 (1)	757 (1)	260 (0)
Education	11,519 (2)	5803 (1)	5716 (5)	34,843 (25)	15,505 (18)	19,338 (34)
Health and social services	26,715 (5)	22,257 (6)	4458 (4)	76,358 (54)	55,926 (66)	20,432 (36)
Missing	1,271 (0)	1,129 (0)	142 (0)	3,484 (2)	3,012 (4)	472 (1)
Health-related characteristics						
Psychiatric morbidity^b^	9346 (2)	7785 (2)	1561 (1)	3341 (2)	2497 (3)	844 (1)
Somatic morbidity^c^	123,643 (24)	96,505 (24)	27,138 (22)	38,893 (27)	24,049 (29)	14,844 (26)
Work disability factors^a^						
No sickness absence (SA)	488,859 (94)	372,587 (93)	116,272 (95)	129,352 (91)	76,150 (91)	53,202 (93)
Short-term SA (0–90 days)	28,191 (5)	23,282 (6)	4909 (4)	10,441 (7)	6765 (8)	3676 (6)
Long-term SA (> 90 days)	5087 (1)	4271 (1)	816 (1)	1653 (1)	1199 (1)	454 (1)

^a^In 2009

^b^Defined by ICD-10 codes F00–99

^c^Defined by ATC-codes A10, N03A excluding mood stabilizers; and/or any ICD-10 code, excluding codes F00–99, O80 and R00–99

### Statistical analysis

Statistically significance of differences between groups (Table 1) was evaluated by *χ*^2^ tests. Multivariate analyses were conducted with Cox regression models of time to first SA due to CMDs (any length and LTSA, respectively). Results are presented as Hazard Ratios (HR) with 95% confidence intervals (CIs). We assessed person-years at risk by totaling the years that the individuals were alive and living in Sweden during the follow-up period. The entry date was defined as January 1st, 2010, and the exit date as the date of first outcome, date of death, date of DP, date of emigration, or the end of follow-up (December 31st, 2016). We present the crude estimates and the estimates adjusted for all covariates described above. Analyses were conducted in SAS 9.4 (SAS Institute Inc., Cary, NC).

#### Sensitivity analyses

As both SA and CMDs are more common in women [4, 19, 28, 29], we carried out sex-stratified analyses (presented as supplementary tables). In additional sensitivity analyses, we excluded 18,502 individuals with mental disorder in 2009 and re-ran the analyses. Here, mental disorders were defined as either being sickness absent with a psychiatric diagnosis (ICD-10: F00–99) or being treated in inpatient or specialized outpatient care with a psychiatric diagnosis in the same year. Last, in separate analyses we compared characteristics for those excluded due to missing data on occupational class with the individuals included in the study.

## Results

Sociodemographic characteristics of the study population are presented in Table 1. The majority of individuals worked in the private sector (79% vs 21%). Moreover, those working in the private sector were slightly younger, more often males, had a lower educational level, and were more often living in a big city area than employees in the public sector (*p* < 0.001). Within the private sector, 77% were manual workers (compared to 60% of those working in the public sector). Non-manual workers in the private sector were more likely to be females and to have college or university degree, when compared to manual workers in the same sector.

Table 2 presents work- and health-related characteristics for the cohort, stratified by sector of employment and occupational class. In the private sector, a large proportion (30%) worked within industry occupations (30%), followed by service occupations (29%). Public sector employees on the other hand were more likely to work in education (54%). Of them especially manual workers had slightly higher levels of psychiatric and somatic morbidity and SA at baseline compared to workers in the private sector.

Sector of employment was associated with SA due to CMDs (Table 3), such that public sector employees had an elevated risk when compared with individuals employed in the private sector [adjusted HR (aHR) for SA (any number of days): 1.31 (95% CI 1.29–1.33) and for LTSA: 1.29 (95% CI 1.26–1.32)]. Overall, manual workers had a slightly elevated risk for SA due to CMDs compared to non-manual workers. When stratifying the analyses by sector of employment, manual workers in the public sector had a particularly increased risk for SA due to CMDs when compared to non-manual workers in the same sector (aHR for SA (any number of days): 1.13 (95% CI 1.09–1.18) and for LTSA: 1.11 (95% CI 1.05–1.17)). These patterns were not observed in the private sector.

**Table 3:** Association between sector of employment, occupational class, and sickness absence (SA) due to common mental disorder (CMDs) in employees, aged 19–29 years residing in Sweden in 2009. Hazard ratios (HRs) with 95% confidence intervals (CIs)

	Sickness absence (any length) due to CMDs			Long-term sickness absence (> 90 days) due to CMDs		
	*n* (rate per 100,000 person-years)	Model 1^a^	Model 2^b^	*n* (rate per 100,000 person-years)	Model 1^a^	Model 2^b^
Sector of employment						
Private sector	47,638 (137.5)	1 (REF)	1 (REF)	23,857 (67.3)	1 (REF)	1 (REF)
Public sector	21,482 (235.3)	1.72 (1.69–1.75)	1.31 (1.29–1.33)	11,032 (116.3)	1.73 (1.70–1.77)	1.29 (1.26–1.32)
Occupational class						
Non-manual workers	18,159 (154.3)	1 (REF)	1 (REF)	9710 (80.5)	1 (REF)	1 (REF)
Manual workers	50,961 (159.3)	1.03 (1.01–1.05)	1.04 (1.02–1.06)	25,179 (76.6)	0.95 (0.93–0.97)	0.99 (0.96–1.02)
Private sector						
Non-manual workers	10,718 (133.2)	1 (REF)	1 (REF)	5782 (70.4)	1 (REF)	1 (REF)
Manual workers	36,920 (138.8)	1.04 (1.02–1.06)	1.04 (1.01–1.06)	18,075 (66.4)	0.94 (0.91–0.97)	0.97 (0.94–1.00)
Public sector						
Non-manual workers	7441 (200.1)	1 (REF)	1 (REF)	3928 (102.2)	1 (REF)	1 (REF)
Manual workers	14,041 (259.6)	1.30 (1.26–1.34)	1.13 (1.09–1.18)	7104 (125.9)	1.23 (1.19–1.28)	1.11 (1.05–1.17)

^a^Model 1: crude

^b^Model 2: adjusted for birth year, sex, education, family situation, type of residential area, long-term sickness absence, and health care due to psychiatric or somatic morbidity in 2009

Tables 4 and 5 show the HRs for SA due to CMDs by occupational class and employment branch in private and public sector employees, respectively. For private sector employees, in both manual and non-manual workers, those employed in education and health and social services evidenced the highest rates and risks of SA due to CMDs. In manual workers, when compared to employees in industry occupations, highest HRs for any SA were observed in health and social services [aHR: 1.43 (95% CI 1.37–1.50), and for LTSA in education (aHR: 1.45 (95% CI 1.31–1.61)]. Construction workers (a majority of which were males) on the other hand evidenced the lowest risk for both outcomes. In non-manual workers, those employed in education had the highest risk of SA (any days) [aHR: 1.45 (95% CI 1.33–1.59)] as well as of LTSA [aHR: 1.35 (95% CI 1.20–1.53)] when compared to non-manual workers in industry occupations (Table 4). Similar tendencies were observed for public sector employees (Table 5), but due to small numbers, adjusted estimates were not statistically significant.

**Table 4:** Hazard Ratios (HR) with 95% confidence intervals (CIs) for SA and LTSA due to CMDs, respectively, by occupational class and employment branch, in young individuals employed in the private sector, aged 19–29 years residing in Sweden in 2009

	Industry occupations	Service occupations	Wholesale and retail trade; Hotel, food service activities	Transportation	Construction	Education	Health and social services
Sickness absence due to common mental disorders (CMDs)							
Manual workers							
*n* (rate per 10,000 person-years)	4893 (107.8)	8901 (150.5)	13,123 (149.8)	2745 (120.6)	2561 (79.3)	912 (243.7)	3670 (257.9)
Model 1^a^	1 (REF)	1.40 (1.35–1.45)	1.40 (1.35–1.44)	1.12 (1.07–1.17)	0.74 (0.70–0.77)	2.28 (2.12–2.45)	2.41 (2.31–2.52)
Model 2^b^	1 (REF)	1.14 (1.10–1.18)	1.06 (1.03–1.10)	1.16 (1.11–1.21)	0.90 (0.86–0.95)	1.41 (1.31–1.51)	1.43 (1.37–1.50)
Non-manual workers							
*n* (rate per 10,000 person-years)	1277 (105.6)	5822 (129.2)	1582 (144.8)	410 (136.9)	289 (108.0)	771 (207.1)	562 (194.5)
Model 1^a^	1 (REF)	1.23 (1.15–1.30)	1.37 (1.28–1.48)	1.30 (1.16–1.45)	1.02 (0.90–1.16)	1.97 (1.80–2.15)	1.85 (1.67–2.04)
Model 2^b^	1 (REF)	1.13 (1.06–1.20)	1.07 (0.99–1.15)	1.09 (0.98–1.22)	1.05 (0.93–1.20)	1.45 (1.33–1.59)	1.24 (1.12–1.37)
Long-term sickness absence due to CMDs							
Manual workers							
*n* (rate per 10,000 person-years)	2308 (49.9)	4493 (74.1)	6458 (72.0)	1312 (56.5)	1178 (36.0)	468 (120.2)	1794 (120.5)
Model 1^a^	1 (REF)	1.49 (1.42–1.57)	1.45 (1.38–1.52)	1.13 (1.06–1.21)	0.72 (0.67–0.77)	2.42 (2.20–2.68)	2.43 (2.29–2.59)
Model 2^b^	1 (REF)	1.20 (1.14–1.26)	1.10 (1.05–1.16)	1.18 (1.10–1.26)	0.92 (0.85–0.98)	1.45 (1.31–1.61)	1.39 (1.30–1.48)
Non-manual workers							
*n* (rate per 10,000 person-years)	715 (58.2)	3122 (67.9)	865 (77.4)	212 (69.4)	164 (60.3)	415 (107.9)	288 (96.0)
Model 1^a^	1 (REF)	1.17 (1.08–1.27)	1.33 (1.21–1.47)	1.19 (1.02–1.39)	1.03 (0.87–1.23)	1.86 (1.65–2.10)	1.65 (1.44–1.89)
Model 2^b^	1 (REF)	1.08 (1.00–1.17)	1.04 (0.94–1.15)	1.00 (0.86–1.17)	1.07 (0.90–1.27)	1.35 (1.20–1.53)	1.10 (0.95–1.26)

^a^Model 1: crude

^b^Model 2: adjusted for birth year, sex, education, family situation, type of residential area, long-term sickness absence, and health care due to psychiatric or somatic morbidity in 2009

**Table 5:** Hazard Ratios (HR) with 95% confidence intervals (CIs) for SA and LTSA due to CMDs, respectively, by occupational class and employment branch, in young individuals employed in the public sector, aged 19–29 years residing in Sweden in 2009

	Industry occupations	Service occupations	Wholesale and retail trade; Hotel, food service activities	Transportation	Construction	Education	Health and social services
Sickness absence due to common mental disorders (CMDs)							
Manual workers							
*n* (rate per 10,000 person-years)	15 (106.1)	925 (167.7)	30 (199.6)	11 (159.5)	57 (111.7)	2,376 (236.3)	10,164 (284.8)
Model 1^a^	1 (REF)	1.59 (0.95–2.64)	1.89 (1.02–3.51)	1.51 (0.69–3.28)	1.05 (0.60–1.86)	2.25 (1.35–3.73)	2.71 (1.64–4.50)
Model 2^b^	1 (REF)	1.17 (0.70–1.94)	1.02 (0.55–1.90)	1.10 (0.50–2.39)	0.99 (0.56–1.75)	1.27 (0.76–2.11)	1.45 (0.87–2.41)
Non-manual workers							
*n* (rate per 10,000 person-years)	9 (170.6)	1777 (162.8)	3 (290.0)	20 (227.2)	27 (154.8)	2599 (208.7)	2941 (222.9)
Model 1^a^	1 (REF)	0.95 (0.50–1.84)	1.70 (0.46–6.27)	1.33 (0.61–2.93)	0.91 (0.43–1.93)	1.23 (0.64–2.36)	1.31 (0.68–2.52)
Model 2^b^	1 (REF)	0.99 (0.52–1.91)	1.59 (0.43–5.86)	1.35 (0.62–2.98)	0.92 (0.43–1.96)	1.11 (0.58–2.13)	1.09 (0.57–2.10)
Long-term sickness absence due to CMDs							
Manual workers							
*n* (rate per 10,000 person-years)	7 (48.6)	484 (85.4)	14 (90.9)	5 (71.2)	29 (56.0)	1,213 (116.3)	5143 (137.6)
Model 1^a^	1 (REF)	1.76 (0.84–3.71)	1.88 (0.76–4.65)	1.47 (0.47–4.63)	1.15 (0.51–2.63)	2.41 (1.15–5.06)	2.85 (1.36–5.98)
Model 2^b^	1 (REF)	1.32 (0.62–2.78)	1.02 (0.41–2.52)	1.10 (0.35–3.45)	1.15 (0.50–2.63)	1.38 (0.66–2.91)	1.54 (0.73–3.23)
Non-manual workers							
*n* (rate per 10,000 person-years)	2 (36.1)	910 (81.1)	2 (185.7)	17 (189.8)	14 (78.7)	1,401 (108.7)	1548 (113.1)
Model 1^a^	1 (REF)	2.25 (0.56–9.00)	5.18 (0.73–36.76)	5.29 (1.22–22.84)	2.18 (0.50–9.58)	3.03 (0.76–12.09)	3.14 (0.79–12.55)
Model 2^b^	1 (REF)	2.44 (0.61–9.75)	4.96 (0.70–35.24)	5.90 (1.36–25.58)	2.34 (0.53–10.29)	2.79 (0.70–11.15)	2.67 (0.67–10.71)

^a^Model 1: crude

^b^Model 2: adjusted for birth year, sex, education, family situation, type of residential area, long-term sickness absence, and health care due to psychiatric or somatic morbidity in 2009

In sensitivity analyses stratified by sex (see Supplementary Tables 1 and 2), we found sex differences in employment branches. For instance, in the private sector, 72% of the women worked in service, wholesale and retail trade, compared to 33% of the men. A large proportion of men was found in industry and construction occupations (see Supplementary Table 1). In both the private and public sector, women were also more likely to work in health and social services compared to men. During the follow-up women had markedly higher rates of SA and LTSA due to CMDs compared to men (see Supplementary Table 2). Moreover, a significant association between occupational class and SA was seen for women, but not for men [aHR for SA (any days) in women: 1.05 (95% CI 1.03–1.08), and in men: 1.01 (95% CI 0.97–1.06)].

We also carried out sensitivity analyses excluding individuals with a treated mental disorder at baseline (see Supplementary Table 3). Results from these analyses were similar to the main analyses.

Analyses not shown revealed that individuals excluded due to missing on occupational class were more likely to be younger, male, born abroad and to possess less education compared to those included in the study. They had slightly lower rates of SA due to CMDs compared to the final study sample.

## Discussion

### Key findings

In this population-based cohort study of 663,583 young employees in Sweden, we found that sector of employment was associated with SA due to CMDs, such that public sector workers had an elevated risk when compared with private sector employees. Moreover, manual workers had a slightly elevated risk for SA due to CMDs compared to non-manual workers. Within the private sector, in both manual and non-manual workers, those employed in education and health and social services evidenced the highest rates and risks of SA due to CMDs. The lowest risks of both outcomes were seen in construction workers. Similar tendencies were also observed in public sector employees.

### Findings in relation to other studies

In our study, 10% of the young adults were sickness absent due to CMDs during the follow-up period (Table 3). The proportion was slightly higher (15%) in public sector employees, who had a higher risk of both short- and long-term SA due to CMDs compared to those working in the private sector. These findings are in line with some earlier studies, demonstrating a higher prevalence of all-cause SA in the public sector [19]. To the best of our knowledge, our study is the first to also examine LTSA due to CMDs in the young working population. Our study showed that in general, manual workers had a higher risk compared to non-manual workers. These findings, which have been confirmed in earlier studies [21–23] were observed in both sectors of employment.

When we further analyzed type of employment branch and SA due to CMDs, private sector employees in education and health and social services stood out as having highest rates of SA due to CMDs (Table 4). Moreover, among manual workers, those working in service occupations also had an elevated risk of SA when compared with employees in industry occupations, which is consistent with earlier research [19]. In public sector employees, occurrence of SA due to CMDs was quite low when stratified by employment branch groups. Nevertheless, those employed in health and social services had highest rates of SA due to CMDs, which is in accordance with some prior studies [20, 21].

The higher rate of SA due to CMDs found among young public sector employees may have various underlying reasons. First of all, we observed a higher psychiatric and somatic morbidity in public sector employees, which may explain the higher SA rates. Still, analyses have been adjusted for previous specialized health care. We also noticed higher SA rates at baseline in public sector employees. Occupational class differences in SA in general [21, 24, 30–32] and due to mental disorders [21, 31] are known. In our study, manual workers had a higher risk of SA due to CMDs compared to non-manual workers, both in the private and public sector. One explanation for the socioeconomic inequalities in depression-related SA is that manual workers may be exposed to less favorable working conditions [22], such as heavy physical work demands and uncomfortable working positions, all of which have been identified as a predictor of SA. Here, it is also important to consider that the association between occupational class and SA due to CMDs may vary between those who seek treatment and those who do not. It has been shown that socioeconomic status play a part in treatment-seeking, even in Sweden, a country with universal access to healthcare [33]. The effect of socioeconomic status on the risk of sickness absence due to CMDs is, therefore, multifaceted. Here pathways to early marginalization might be driven by both health selection and social causation processes, which are likely to be strongly intertwined [34]. As CMDs are often characterized by an early age of onset, problems might already arise in adolescence and young adulthood when affected individuals may not fully develop knowledge and competencies or psychological and cognitive capabilities [35]. Such competencies and capabilities are necessary for achieving certain educational and occupational levels. Particularly the accumulation of disadvantages over the life course may take a long-term toll on health through a process that puts people already facing serious adversities at risk for continued exposure to further adverse circumstances [36]. Individuals exposed to social inequalities and marginalization at the labor market may be trapped in a self-perpetuating cycle involving stressful life and workplace circumstances, poor mental health and restrictions in social and occupational functioning leading to long periods of work disability [36, 37]. Strategies targeting adolescents with poor psychosocial life conditions (i.e., before entrance in the labor market) are, therefore, advisable to prevent early labor market marginalization in young adults.

Moreover, differences in psychosocial work environment may explain these findings as it has consistently been shown to be of great importance for mental health. Psychosocial work stressors that have been pointed out as risk factors for CMDs include job strain, low decision latitude, low social support, high psychological demands, effort–reward imbalance, and high job insecurity. In addition, there may be other factors contributing to the socioeconomic differences in SA due to mental disorders. For example, comorbid mental and somatic disorders are more common in lower SES groups [11, 38], and similar tendencies were also observed in our study. In addition, the socioeconomic gradient in overall sickness absence could also be attributed to health behaviors, which tend to be more detrimental in manual occupations.

With respect to differences in employment branches, higher SA levels have been observed in individuals employed in the health and social work sector, which was also demonstrated in our study on young employees. These findings have among others been explained by the unfavorable psychosocial and physical work conditions more present in these branches [39].

### Strengths and limitations

The strengths of this study include the longitudinal population-based design, use of national registers with high completeness and validity, and practically no loss to follow-up. Moreover, with the large sample size, we were able to conduct detailed analyses of different employment branches, and ability to adjust for a range of important confounders. Still a number of important limitations need to be considered when interpreting the study results. First, the register does not contain information about the first 14 days of the SA spell, as these are usually paid by the employer. Therefore, those with SA < 14 days will be misclassified as not having the outcome. This potential outcome misclassification could lead to an underestimation of the studied associations. Second, with respect to comorbidity, there is a potential risk of unmeasured residual confounding as morbidity was only measured by specialized health care. The lack of data on occupational class throughout the entire follow-up period might have led to over- or underestimation of the reported estimates. Last, we did not have information on factors reflecting the work environment. This may be especially relevant regarding the psychosocial work environment, as this may be of importance for the studied associations.

## Conclusion

In conclusion, this study of young employees in Sweden suggests that public sector workers, and those working in manual occupations are particularly at risk for SA due to CMDs. Within different employment branches, those working in health care and education were identified as having an elevated risk of SA due to CMDs. These findings should be considered when identifying high-risk groups for SA in the young working population.

## Supplementary Information

Below is the link to the electronic supplementary material.

Supplementary file1 (DOCX 28 KB)
