# Supplementary material for: Sickness absence due to common mental disorders in young employees in Sweden: are there differences in occupational class and employment sector?
Source: Soc Psychiatry Psychiatr Epidemiol. 2021 Aug 12;57(5):1097–106. doi: 10.1007/s00127-021-02152-3 (PMC9042979; doi:10.1007/s00127-021-02152-3)
Supplement: Supplementary file 1 — Supplementary file1 (DOCX 28 KB) [file 127_2021_2152_MOESM1_ESM.docx]

***Supplementary Table 1.*** *Selected characteristics stratified by sex and sector of employment, in individuals aged 19-29 years old residing in Sweden in 2009.*

|  | **Private sector** | | **Public sector** | |
| --- | --- | --- | --- | --- |
| **Cohort characteristics^1^** | **Women** | **Men** | **Women** | **Men** |
| All, (n, row percent) | 221,367 (100) | 300,770 (100) | 105 881 | 35 565 |
| ***Sociodemographic factors*** |  |  |  |  |
| Mean age (years, SD) | 24.4 (3.1) | 24.8 (2.9) | 25.1 (3.0) | 25.4 (2.9) |
| Country of birth |  |  |  |  |
| Sweden | 199,200 (90) | 268,237 (89) | 94,138 (89) | 30,971 (87) |
| Other Nordic | 1,604 (1) | 1,651 (1) | 721 (1) | 179 (1) |
| EU25 (except Denmark, Finland and Sweden) | 3,191 (1) | 4,231 (1) | 1,374 (1) | 776 (2) |
| The rest of the world | 17,353 (8) | 26,629 (9) | 9,639 (9) | 3,628 (10) |
| Missing | 19 (0) | 22 (0) | 9 (0) | 11 (0) |
| Education (years) |  |  |  |  |
| Compulsory school (<9) | 14,858 (7) | 29,828 (10) | 5,113 (5) | 1,922 (5) |
| High school (10–12) | 131,665 (59) | 201,088 (67) | 50,238 (47) | 15,831 (45) |
| College or university (>12) | 73,754 (33) | 67,248 (22) | 50,215 (47) | 17,548 (49) |
| Missing | 1,090 (0) | 2,606 (1) | 315 (0) | 264 (1) |
| Family situation |  |  |  |  |
| Married/living with partner without children^2^ | 8,384 (4) | 8,239 (3) | 4,955 (5) | 1,313 (4) |
| Married/living with partner with children^2^ | 35,014 (16) | 37,922 (13) | 25,544 (24) | 4,384 (12) |
| Single/divorced/separated/widowed without children^2^ | 150,765 (68) | 232,033 (77) | 63,831 (60) | 27,284 (77) |
| Single/divorced/separated/widowed with children^2^ | 6,527 (3) | 972 (0) | 4,230 (4) | 101 (0) |
| Children (≤20 years old)^2^ | 20,677 (9) | 21,604 (7) | 7,321 (7) | 2,483 (7) |
| Type of residential area |  |  |  |  |
| Big city area | 102,131 (46) | 119,397 (40) | 35,026 (33) | 13,530 (38) |
| Intermediate (>90,000 inhabitants) | 74,040 (33) | 105,824 (35) | 41,895 (40) | 13,735 (39) |
| Small (rural municipalities) | 45,196 (20) | 75,549 (25) | 28,960 (27) | 8,300 (23) |
| ***Work-related factors*** |  |  |  |  |
| Employment branch |  |  |  |  |
| Industry occupations | 20,528 (9) | 53,682 (18) | 82 (0) | 208 (1) |
| Service occupations | 71,916 (32) | 48,122 (16) | 13,562 (13) | 11,408 (32) |
| Wholesale and retail trade; Accommodation and food service activities | 88,944 (40) | 52,427 (17) | 196 (0) | 47 (0) |
| Transportation | 7,599 (3) | 28,335 (9) | 120 (0) | 121 (0) |
| Construction | 2,744 (1) | 45,955 (15) | 285 (0) | 732 (2) |
| Education | 8,278 (4) | 1,439 (0) | 25,721 (24) | 9,122 (26) |
| Health and social services | 20,689 (9) | 5,086 (2) | 63,430 (60) | 12,928 (36) |
| Missing | 669 (0) | 530 (0) | 2,485 (2) | 999 (3) |
| ***Health-related factors*** |  |  |  |  |
| Psychiatric morbidity^3^ | 5,043 (2) | 3,728 (1) | 2,717 (3) | 624 (2) |
| Somatic morbidity^4^ | 60,546 (27) | 51,181 (17) | 31,728 (30) | 7,165 (20) |
| ***Work disability factors (from LISA)*** |  |  |  |  |
| No sickness absence (SA) | 202,930 (92) | 222,395 (74) | 95,062 (90) | 34,290 (96) |
| Short-Term SA (0-90 days) | 15,613 (7) | 11,194 (4) | 9,381 (9) | 1,060 (3) |
| Long-term SA (>90 days) | 2,824 (1) | 1,987 (1) | 1,438 (1) | 215 (1) |

^1^ In 2009 ^2^ Living at home ^3^ Defined by ICD-10 codes F00-99

^4^ Defined by ATC-codes A10, N03A excluding mood stabilizers; and/or any ICD-10 code, excluding codes F00-99, O80 and R00-99

***Supplementary Table 2.*** *Associations between sector of employment, occupational class, and sickness absence due to common mental disorders, 2010-2016 in employees, aged 19-29 years old residing in Sweden in 2009. Hazard ratios (HRs) with 95% confidence intervals (CIs),* ***stratified by sex.***

|  | **Women** | | | **Men** | | |
| --- | --- | --- | --- | --- | --- | --- |
|  | **n (rate per 100,000 person-years)** | **Model 1^a^** | **Model 2^b^** | **n (rate per 100,000 person-years)** | **Model 1^a^** | **Model 2^b^** |
|  | **Sickness absence due to common mental disorders** | | | | | |
| **Sector of employment** |  |  |  |  |  |  |
| Private sector | 30,814 (214.6) | 1 (REF) | 1 (REF) | 16,824 (83.0) | 1 (REF) | 1 (REF) |
| Public sector | 19,171 (284.2) | 1.33 (1.31-1.35) | 1.31 (1.28-1.33) | 2,311 (97.0) | 1.17 (1.12-1.22) | 1.32 (1.26-1.38) |
| **Occupational class, all** |  |  |  |  |  |  |
| Non-manual workers | 14,179 (224.2) | 1 (REF) | 1 (REF) | 3,980 (73.2) | 1 (REF) | 1 (REF) |
| Manual workers | 35,806 (242.3) | 1.08 (1.06-1.10) | 1.05 (1.03-1.08) | 15,155 (88.0) | 1.20 (1.16-1.24) | 1.01 (0.97-1.06) |
| **Occupational class, private sector** |  |  |  |  |  |  |
| Non-manual workers | 7,572 (206.1) | 1 (REF) | 1 (REF) | 3,146 (72.0) | 1 (REF) | 1 (REF) |
| Manual workers | 23,242 (217.6) | 1.06 (1.03-1.08) | 1.05 (1.02-1.08) | 13,678 (86.0) | 1.19 (1.15-1.24) | 1.01 (0.96-1.05) |
| **Occupational class, public sector** |  |  |  |  |  |  |
| Non-manual workers | 6,607 (249.3) | 1 (REF) | 1 (REF) | 834 (78.0) | 1 (REF) | 1 (REF) |
| Manual workers | 12,564 (306.8) | 1.23 (1.20-1.27) | 1.12 (1.08-1.18) | 1,477 (112.4) | 1.44 (1.32-1.57) | 1.20 (1.07-1.33) |
|  |  |  |  |  |  |  |
|  | **Long-term sickness absence due to common mental disorders** | | | | | |
| **Sector of employment** |  |  |  |  |  |  |
| Private sector | 15,881 (106.9) | 1 (REF) | 1 (REF) | 7,976 (38.8) | 1 (REF) | 1 (REF) |
| Public sector | 9,968 (141.2) | 1.32 (1.29-1.36) | 1.27 (1.24-1.31) | 1,064 (43.9) | 1.13 (1.06-1.21) | 1.26 (1.18-1.35) |
| **Occupational class, all** |  |  |  |  |  |  |
| Non-manual workers | 7,682 (117.2) | 1 (REF) | 1 (REF) | 2,028 (36.8) | 1 (REF) | 1 (REF) |
| Manual workers | 18,167 (118.2) | 1.01 (0.98-1.04) | 1.01 (0.98-1.04) | 7,012 (40.1) | 1.09 (1.03-1.14) | 0.91 (0.86-0.96) |
| **Occupational class, private sector** |  |  |  |  |  |  |
| Non-manual workers | 4,130 (108.8) | 1 (REF) | 1 (REF) | 1,652 (37.4) | 1 (REF) | 1 (REF) |
| Manual workers | 11,751 (106.2) | 0.98 (0.94-1.01) | 0.99 (0.96-1.04) | 6,324 (39.2) | 1.05 (0.99-1.10) | 0.88 (0.83-0.94) |
| **Occupational class, public sector** |  |  |  |  |  |  |
| Non-manual workers | 3,552 (128.7) | 1 (REF) | 1 (REF) | 376 (34.7) | 1 (REF) | 1 (REF) |
| Manual workers | 6,416 (149.2) | 1.16 (1.11-1.21) | 1.08 (1.02-1.15) | 688 (51.4) | 1.48 (1.30-1.68) | 1.21 (1.03-1.43) |

^a^ Model 1: Crude

^b^ Model 2: Adjusted for birth year, education, family situation, type of residential area, long-term sickness absence, and health care due to psychiatric or somatic morbidity in 2009

***Supplementary Table 3.*** *Associations between sector of employment, occupational class, and sickness absence (SA) due to common mental disorder (CMDs) in employees, aged 19-29 years old residing in Sweden in 2009. Hazard ratios (HRs) with 95% confidence intervals (CIs). Excluded are individuals with a mental disorder in 2009*.*

|  | **Sickness absence (any length) due to CMDs** | | | **Long-term sickness absence (>90 days) due to CMDs** | | |
| --- | --- | --- | --- | --- | --- | --- |
|  | **n (rate per 100,000 person-years)** | **Model 1^a^** | **Model 2^b^** | **n (rate per 100,000 person-years)** | **Model 1^a^** | **Model 2^b^** |
| **Sector of employment** |  |  |  |  |  |  |
| Private sector | 43,458 (128.2) | 1 (REF) | 1 (REF) | 21,336 (61.6) | 1 (REF) | 1 (REF) |
| Public sector | 19,410 (219.0) | 1.72 (1.69-1.75) | 1.32 (1.29-1.34) | 9,770 (106.4) | 1.73 (1.69-1.77) | 1.28 (1.25-1.32) |
|  |  |  |  |  |  |  |
| **Occupational class, all** |  |  |  |  |  |  |
| Non-manual workers | 16,669 (144.4) | 1 (REF) | 1 (REF) | 8,756 (74.1) | 1 (REF) | 1 (REF) |
| Manual workers | 46,199 (148.0) | 1.02 (1.01-1.04) | 1.05 (1.03-1.07) | 22,350 (69.9) | 0.94 (0.92-0.97) | 1.00 (0.97-1.03) |
|  |  |  |  |  |  |  |
|  |  |  |  |  |  |  |
| Occupational class, private sector |  |  |  |  |  |  |
| Non-manual workers | 9,868 (124.8) | 1 (REF) | 1 (REF) | 5,239 (65.0) | 1 (REF) | 1 (REF) |
| Manual workers | 33,590 (129.3) | 1.03 (1.01-1.06) | 1.05 (1.02-1.07) | 16,097 (60.6) | 0.93 (0.90-0.96) | 0.97 (0.94-1.01) |
|  |  |  |  |  |  |  |
| Occupational class, public sector |  |  |  |  |  |  |
| Non-manual workers | 6,801 (186.7) | 1 (REF) | 1 (REF) | 3,517 (93.6) | 1 (REF) | 1 (REF) |
| Manual workers | 12,609 (241.5) | 1.30 (1.26-1.34) | 1.13 (1.09-1.18) | 6,253 (115.2) | 1.23 (1.18-1.28) | 1.11 (1.05-1.18) |

^*^ Defined as SA, any length, with a psychiatric diagnosis, or treatment in inpatient or specialized outpatient care with a psychiatric diagnosis

^a^ Model 1: Crude

^b^ Model 2: Adjusted for birth year, sex, education, family situation, type of residential area, long-term sickness absence, and health care due to somatic morbidity in 2009
